# Reassessment of *FBN1* variants of uncertain significance using updated ClinGen guidance for PP1/BS4 and PP4 criteria

**DOI:** 10.1038/s41431-025-01826-9

**Published:** 2025-04-01

**Authors:** Ju Hyeon Shin, Young-gon Kim, Shin Yi Jang, June Huh, Duk-Kyung Kim, Jong-Won Kim, Ja-Hyun Jang, Taek Kyu Park, Mi-Ae Jang

**Affiliations:** 1 Department of Laboratory Medicine and Genetics, Samsung Medical Center, Sungkyunkwan University School of Medicine, Seoul, South Korea; 2 Division of Cardiology, Department of Medicine, Heart Vascular Stroke Institute, Samsung Medical Center, Sungkyunkwan University School of Medicine, Seoul, South Korea; 3 Division of Cardiology, Department of Pediatrics, Adult Congenital Heart Disease Clinic, Heart Vascular Stroke Institute, Samsung Medical Center, Sungkyunkwan University School of Medicine, Seoul, South Korea; 4 Division of Cardiology, Department of Internal Medicine, Samsung Changwon Hospital, Sungkyunkwan University School of Medicine, Changwon-si, South Korea

**Keywords:** Genetic testing, Cardiovascular diseases

## Abstract

Marfan syndrome (MFS) is a genetic disorder caused by an *FBN1* variant and is diagnosed based on the revised Ghent criteria, which incorporate clinical manifestations and genetic testing. Up-to-date *FBN1* variant interpretation is crucial for proper diagnosis and management of MFS; however, some *FBN1* variants of uncertain significance (VUSs) remain inconclusive despite applying Clinical Genome Resource (ClinGen) *FBN1*-specific guideline. Recently, the ClinGen guidance for PP1/BS4 co-segregation and PP4 phenotype specificity criteria (new PP1/PP4 criteria) were released. Here, we performed reassessment of *FBN1* VUSs using these new PP1/PP4 criteria. *FBN1* VUSs collected from December 2015 to April 2024 were reassessed according to the ClinGen *FBN1*-specific guideline and new PP1/PP4 criteria. Medical records and previous studies were reviewed to evaluate the phenotype-specificity of evidence based on the revised Ghent criteria. Collectively, 927 patients with suspected MFS underwent *FBN1* sequencing and 72 VUSs were detected. When applying the *FBN1*-specific guideline only, of 72 VUSs, 29 (40.3%) were reclassified as pathogenic variants (PVs) or likely PVs (LPVs). When additionally applying the new PP1/PP4 criteria, 16 (37.2%) of the remaining 43 VUSs were reclassified as LPVs. After reassessing *FBN1* VUSs according to the new PP1/PP4 criteria, the rate of reclassification from VUS to PV/LPV significantly increased from 40.3% to 62.5%. The new PP1/PP4 criteria provide sufficient evidence for evaluating the pathogenicity of *FBN1* variants detected in MFS patients fulfilling the revised Ghent criteria and will be helpful in clinical analysis.

## Introduction

Marfan syndrome (MFS) is a hereditary connective tissue disorder involving multiple organs, including the cardiovascular, ocular, and skeletal systems [1]. Because MFS patients are at increased risk for aortic dissection, early and accurate diagnosis is important. According to the revised Ghent criteria, MFS can be diagnosed based on characteristic clinical manifestations such as aortic root dilatation or dissection, ectopia lentis, or several systemic features [2]. In addition, causative *FBN1* variants were detected in about 91–93% of MFS patients fulfilling the Ghent criteria [3, 4]. Considering the high diagnostic yield and importance of *FBN1* variants in MFS, the presence of a pathogenic *FBN1* variant is a diagnostic criterion in Ghent nosology [2, 5]. Consequently, it is crucial for diagnosis and management to evaluate the pathogenicity of any *FBN1* variant found in a patient with suspected MFS.

The American College of Medical Genetics and Association for Molecular Pathology (ACMG/AMP) proposed a guideline for interpretation of sequence variants in 2015, and this guideline is widely accepted in clinical laboratories [6]. However, the ACMG/AMP guideline for the general principles of variant interpretation overlooks the promotion of distinct characteristics of inherited diseases and causative genes. To address this weakness, the Clinical Genome Resource (ClinGen) Variant Curation Expert Panel provided gene-specific variant interpretation guidelines, including the ClinGen *FBN1*-specific guideline, based on the criteria of the ACMG/AMP guideline [7, 8]. By applying the ClinGen *FBN1*-specific guideline released in February 2022, two studies performed the reassessment of variants of uncertain significance (VUSs) [9, 10]. In one study, among 61 VUSs, 38 (62.3%) were reclassified as pathogenic variants (PVs) or likely PVs (LPVs) and 23 (37.7%) retained their initial designation [9]. In the other study, among 26 VUSs, four (15.4%) were reclassified as LPVs and 16 (61.5%) remained as VUSs [10].

New ClinGen guidance for the use of PP1/BS4 co-segregation and PP4 phenotype specificity criteria for sequence variant pathogenicity classification (the new PP1/PP4 criteria) was published in January 2024 [11]. The aforementioned new PP1/PP4 criteria adopt a points-based system proposed by Tavtigian et al. considering the criteria of the ACMG/AMP guideline [12]. This system suggests that the strength of PP4 can be weighted up to 5 points (upper limit of the VUS assignment score) in the case of a genetic disease with a gene-specific phenotype and high diagnostic yield [11, 12]. Reciprocally, PP1 cannot provide additional pathogenic information when PP4 is applied with high strength. As MFS has been known to exhibit locus homogeneity and high diagnostic yield of *FBN1* variants, such a variant detected in a patient with MFS meeting the Ghent criteria would be assigned stronger pathogenic evidence of PP4 [11]. In this regard, an *FBN1* VUS has a chance to be reclassified as a PV/LPV. Here, we performed re-evaluation of *FBN1* VUSs using the ClinGen *FBN1*-specific guideline with the new PP1/PP4 criteria and assessed their utility in the clinical laboratory.

## Materials and methods

### Patients and data collection

Patients with suspected MFS who underwent sequencing testing for *FBN1* at Samsung Medical Center (SMC) from December 2015 to April 2024 were collected. The *FBN1* gene was analyzed using either Sanger sequencing or next-generation sequencing (NGS) for an MFS-related panel (NGS-MFS) or connective tissue disorder-related panel (NGS-CTD). NGS-MFS and NGS-CTD included 18 and 38 genes, respectively (Supplementary Table 1). Briefly, extracted genomic DNA or RNA was amplified and sequenced either using the ABI Prism 3130xl or 3730xl DNA Analyzer (Applied Biosystems, Foster City, CA, USA), the NovaSeq 6000 system (Illumina, San Diego, CA, USA), or the NextSeq 550 system (Illumina) as previously described [9, 13]. All coding exons and their flanking introns (±25 base pairs) were analyzed. Variants were described following HGVS recommendations with reference transcript NM_000138.5. Retrospectively, electronic medical records of patients were reviewed to obtain clinical information, including medical history, family history, and the results of *FBN1* sequencing. Previously reported VUSs were selected and reassessed by applying the ClinGen *FBN1*-specific guideline alone or with the new PP1/PP4 criteria. This study was approved by the Institutional Review Board (IRB) of SMC, Seoul, Korea (approval no. 2024-02-006).

### Applying the new PP1/PP4 criteria

The original ACMG/AMP guideline suggested that PP4 criterion be applied when patient’s phenotype or family history is highly specific for a disease with a single genetic etiology [6]. ClinGen *FBN1*-specific guideline modulated that PP4 can be used if patients fulfill the revised Ghent criteria or any of the family members have a highly specific phenotype [8]. The new PP1/PP4 criteria showed heuristic example of how to apply PP4 in case of MFS [11]. Basically, the stronger PP4 evidence was applied in patients who met the revised Ghent criteria. Even if the clinical data of the patient were insufficient, the stronger PP4 evidence could be applied when a previous study had identified an MFS patient who had the same variant and met the Ghent criteria [11]. We investigated the phenotype data from patients and literature review to distribute them into three categories: (1) old or revised Ghent criteria, (2) consistent but not highly specific phenotype, and (3) highly specific phenotype [2, 5, 8]. According to the ClinGen *FBN1*-specific guideline, ‘consistent but not highly specific phenotype’ was defined as aortic root dilatation (Z-score ≥2 for an individual aged ≥20 years or ≥3 for an individual aged <20 years) or dissection with a ≥7-point systemic score, while ‘highly specific phenotype’ was defined as aortic root dilatation (Z-score ≥2 for an individual aged ≥20 years or ≥3 for an individual aged <20 years) or dissection with simultaneous ectopia lentis [8]. When there were no available phenotype data in detail except for a literature report that a proband fulfilled Ghent criteria, it was included in the category of old or revised Ghent criteria. To preclude the redundancy of applying pathogenic evidence, the proband data were not considered as PS4 evidence if they were already used as stronger PP4 evidence.

The new PP1/PP4 criteria determined the final tier of the variant according to a points-based system, as described by Tavtigian et al. [11, 12]. Pathogenic evidence was converted to points according to its strength (supporting, moderate, strong, and very strong evidence were awarded 1, 2, 4, and 8 points, respectively). Benign evidence was converted in the same manner (supporting, moderate, strong, and very strong evidence were awarded −1, −2, −4, and −8 points, respectively). Exceptionally, the stronger PP4 evidence which was assigned for a variant detected in an MFS patient meeting the Ghent criteria was converted to 5 points according to the new PP1/PP4 criteria [11]. This is because the new PP1/PP4 criteria suggest back-calculating the Bayesian points from the posterior probability of 91%, i.e., diagnostic yield of MFS patients fulfilling the Ghent criteria exhibiting locus homogeneity. Specifically, it was converted from 91% to 6 points of Bayesian points and was capped at 5 points. Furthermore, as PP4 criterion is inseparably coupled to PP1 criterion, PP1 cannot provide additional pathogenic evidence in case of a disease with locus homogeneity and high diagnostic yield. On the contrary, in case of a disease with locus heterogeneity, in addition to back-calculated PP4 points, PP1 can be added based on the inheritance mode of the disorder and numbers of affected family members. Combined PP4 and PP1 points should be capped at 5 points. When PP4 could not be applied because of a phenotype consistent but genetic heterogeneity for MFS (e.g. isolated thoracic aortic aneurysm and dissection), the PP1 was applied instead. The new PP1/PP4 criteria awards points of PP1 criterion with 1 point for each affected family member in disorders with autosomal-dominant inheritance, whereas the ClinGen *FBN1*-specific guideline provides the different strength of PP1 criterion depending on numbers of affected family members such as PP1_strong for ≥5 affected individuals, PP1_moderate for 4 affected individuals, and PP1 for 2–3 affected individuals [8, 11]. The thresholds of the aggregated points for PVs and LPVs were 10 and 6 points, respectively. On the other hand, those for a likely benign variant (LBV) and benign variant were −1 and −7 points, respectively. A variant awarded from 0 to 5 points was categorized as a VUS.

McNemar’s test was performed to compare the reclassification rate of a VUS to a PV/LPV when the new PP1/PP4 criteria were applied or not applied. MedCalc version 22.032 (MedCalc Software Ltd., Acacialaan, Ostend, Belgium) was used, and *P* < 0.05 was considered statistically significant.

### Subclassifying VUS

We evaluated reclassification of VUS subclasses based on the previous paper which demonstrated application of subclassifying VUSs [14]. Initial VUSs were categorized into three subclasses as VUS-high (VUS-H), VUS-mid (VUS-M), and VUS-low (VUS-L) according to the ACMG/AMP criteria at initial diagnosis and our own criteria since there was limited guidance for VUS subclassification. Adopting a point-based system of Tavtigian et al. [12], VUSs were subclassified as follows: a VUS with 4–5 points as VUS-H, with 2–3 points as VUS-M, and less than 2 points as VUS-L. Exceptionally, when there were no available records of variant assessment left in our electronic medical record system, we subclassified it as VUS-unsubclassified (VUS-U).

## Results

### Overall evaluation of the variants

**Fig. 1:**
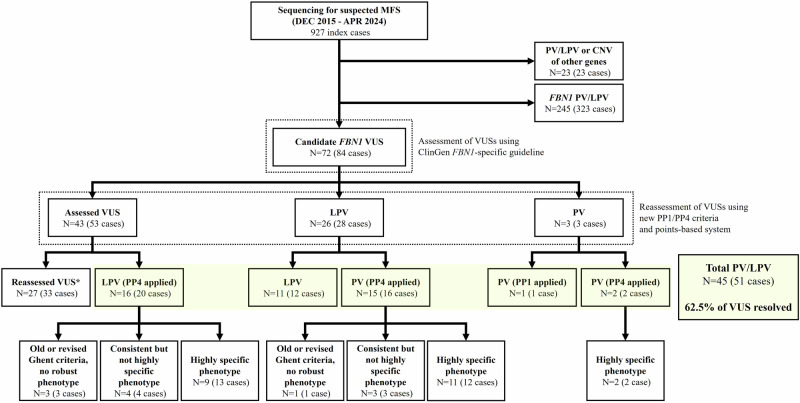
Schematic flow showing the selection of VUSs and the results of variant reassessment in this study. *The reassessed VUS category included 15 VUSs (from 15 cases) and 12 LBVs (from 18 cases) when the points-based system was applied. CNV copy number variation, LBV likely benign variant, LPV likely pathogenic variant, MFS Marfan syndrome, PV pathogenic variant, VUS variant of uncertain significance.

Collectively, 927 probands whose *FBN1* sequencing data were available were included in this study. They underwent either Sanger sequencing (*n* = 657), NGS-MFS (*n* = 161), or NGS-CTD (*n* = 119) while 10 patients were evaluated using both tests (Supplementary Fig. 1). Patient selection and variant classification are shown in Fig. 1. Among 927 cases, 323 had 245 PVs/LPVs of *FBN1* (Supplementary Table 2) and 84 showed 72 VUSs. These 72 VUSs were reassessed using the ClinGen *FBN1*-specific guideline, and 29 variants were reclassified as PVs/LPVs. When the new PP1/PP4 criteria were applied, 16 of the 43 remaining VUSs (37.2%) were reclassified as LPVs. The details of patients’ clinical information, family history, and application of ACMG/AMP criteria are summarized in Tables 1, 2, and Supplementary Table 3. Application of the new PP1/PP4 criteria produced a significant improvement of the reclassification rates of VUSs to PVs/LPVs from 40.3% (29/72) to 62.5% (45/72) (*P* < 0.0001). When stronger PP4 evidence was only applied for highly specific phenotype, the reclassification rates increased from 40.3% to 52.8% (38/72) (*P* = 0.0039).

**Table 1:** Clinical features and ACMG/AMP evidence reassessed with application of the new PP1/PP4 criteria for *FBN1* variants that were assessed as VUSs by ClinGen *FBN1*-specific guideline.

Location	Variant	Age at time of testing (years)	Sex	Aortic root Z-score	Aortic dissection	Ectopia lentis	Systemic score (points)	Revised Ghent criteria	Family study	Literature review	Phenotype data			ACMG/AMP criteria at initial diagnosis	Classification	ACMG/AMP criteria and ClinGen *FBN1*-specific guideline	Reclassification	ACMG/AMP criteria and new PP1/PP4 criteria	Points-based system	Reclassification
											Old or revised Ghent criteria	Consistent but not highly specific phenotype^a^	Highly specific phenotype^b^							
Exon	c.47T>C p.(Leu16Pro)	56	F						3 affected				+^d^	PP2, PM2_P	VUS-M	PP1, PP2, PP4, PM2_P	VUS	PP2, PP4, PM2_P	7	LPV
	c.1679G>A p.(Gly560Asp)	22^c^	F			+				+			+	PM2, PP3, PP5	VUS-H	PP2, PP3, PS4_P, PM2_P	VUS	PP2, PP3, PP4, PM2_P	8	LPV
		17	M	1.54	−	+	5	−												
	c.2504A>T p.(Glu835Val)	23^c^	F	2.95	−	+	12	+					+	PM2	VUS-M	PP2, PP4, PM2_P	VUS	PP2, PP4, PM2_P	7	LPV
	c.2651G>A p.(Gly884Glu)	49^c^	M							+	+			PM2, PP2, PP3, PS4_P	VUS-H	PP2, PP3, PS4_P, PM2_P	VUS	PP2, PP3, PP4, PM2_P	8	LPV
	c.4818_4826del p.(Asp1606_Asp1608del)	32^c^	M	21.1	Focal dissection of the ascending aorta	+	2	+	2 affected				+	PM2, PM4, PP4	VUS-H	PM4, PP1, PP4, PM2_P	VUS	PM4, PP4, PM2_P	8	LPV
	c.4942G>A p.(Asp1648Asn)	40^c^	M							+	+			PM2, PP3, PS4_P	VUS-H	PM1, PP2, PS4_P, PM2_P	VUS	PM1, PP2, PP4, PM2_P	9	LPV
	c.5240_5251del p.(Leu1747_Ser1750del)	57	F		Type A aortic dissection	+	1	+					+	PM2, PM4, PP4	VUS-H	PM4, PP4, PM2_P	VUS	PM4, PP4, PM2_P	8	LPV
		39^c^	M																	
	c.5636G>T p.(Gly1879Val)	12^c^	F	2.39	−	+	7	+	2 affected				+	PM2, PP3	VUS-M	PP1, PP2, PP3, PP4, PM2_P	VUS	PP2, PP3, PP4, PM2_P	8	LPV
		24^c^	M		Type A aortic dissection															
	c.5756G>T p.(Gly1919Val)	37	F	16.6	−	−	8	+		+		+		PP2, PP3, PP4, PS4_P, PM2_P	VUS-H	PP2, PP3, PP4, PS4_P, PM2_P	VUS	PP2, PP3, PP4, PS4_P, PM2_P	9	LPV
	c.6403G>A p.(Asp2135Asn)	61	F	7.93	−	−	7	+				+		PM2_P	VUS-L	PP2, PP4, PM2_P	VUS	PP2, PP4, PM2_P	7	LPV
	c.6704G>T p.(Gly2235Val)	8	M	3.00	−	+	1	+					+	PP2, PP3, PM2_P	VUS-M	PP2, PP3, PP4, PM2_P	VUS	PP2, PP3, PP4, PM2_P	8	LPV
		24^c^	M	5.65	−	NE	8	+												
	c.8012T>C p.(Leu2671Pro)	25^c^	F	4.19	−	−	7	+	3 affected			+		No available records of variant assessment	VUS-U	PP1, PP2, PP3, PP4, PM2_P	VUS	PP2, PP3, PP4, PM2_P	8	LPV
Intron	c.1148−13T>A p.(?)	34^c^	F		Type A aortic dissection	−	7	+	1 affected				+^d^	PM2, PP3	VUS-M	PP3, PP4, PM2_P	VUS	PP3, PP4, PM2_P	7	LPV
	c.5225−3C>G p.(?)	23^c^	M							+	+			PM2, PP3	VUS-M	PP3, PS4_P, PM2_P	VUS	PP3, PP4, PM2_P	7	LPV
	c.6872−14A>G p.(?)	31^c^	M							+			+	PM2, PP3	VUS-M	PS4_M, PP3, PM2_P, PM6_P	VUS	PP3, PP4, PS4_P, PM2_P, PM6_P	9	LPV
	c.7819+5G>A p.(?)	33^c^	F	6.35	−	−	9	+	1 affected	+		+		PM2, PP3	VUS-M	PP3, PP4, PS4_P, PM2_P	VUS	PP3, PP4, PS4_P, PM2_P	8	LPV

*LPV* likely pathogenic variant, *NE* not evaluated, *VUS* variant of uncertain significance, *VUS-H* variant of uncertain significance-high subclass, *VUS-L* variant of uncertain significance-low subclass, *VUS-M* variant of uncertain significance-mid subclass, *VUS-U* variant of uncertain significance-unsubclassified.

^a^Consistent but not highly specific phenotype definition: aortic diameter at the sinus of Valsalva above the indicated Z-score (≥2 for an individual aged ≥20 years or ≥3 for an individual aged <20 years) or aortic root dissection AND systemic score ≥7 points.

^b^Highly specific phenotype definition: aortic diameter at the sinus of Valsalva above the indicated Z-score (≥2 for an individual aged ≥20 years or ≥3 for an individual aged <20 years) or aortic root dissection AND ectopia lentis.

^c^This patient had been reported in the previous study of Samsung Medical Center [9].

^d^Highly specific phenotype was identified in a family member with the same variant: 1) one patient with c.47T>C p.(Leu16Pro) presented an aortic root Z-score of 8.38, ectopia lentis, and a systemic score of 4 points; 2) one patient with c.1148-13T>A p.(?) presented an aortic root Z-score 3.15, ectopia lentis, and a systemic score of 7 points.

**Table 2:** Clinical features and ACMG/AMP evidence reassessed with application of the new PP1/PP4 criteria for *FBN1* variants that were assessed as LPVs by ClinGen *FBN1*-specific guideline.

Location	Variant	Age at time of testing (years)	Sex	Aortic root Z-score	Aortic dissection	Ectopia lentis	Systemic score (points)	Revised Ghent criteria	Family study	Literature review	Phenotype data			ACMG/AMP criteria at initial diagnosis	Classification	ACMG/AMP criteria and ClinGen *FBN1*-specific guideline	Reclassification	ACMG/AMP criteria and new PP1/PP4 criteria	Points-based system	Reclassification
											Old or revised Ghent criteria	Consistent but not highly specific phenotype^a^	Highly specific phenotype^b^							
Exon	c.1884C>G p.(Cys628Trp)	22^c^	M	5.39	−	+	11	+		+			+	PM1, PM2, PP4	VUS-H	PM1_S, PS4_M, PP2, PP4, PM2_P	LPV	PM1_S, PS4_M, PP2, PP4, PM2_P	13	PV
	c.2585G>A p.(Cys862Tyr)	27^c^	M							+			+	No available records of variant assessment	VUS-U	PM1, PM5, PP2, PP3, PS4_P, PM2_P	LPV	PM1, PM5, PP2, PP3, PP4, PM2_P	12	PV
	c.3379G>T p.(Gly1127Cys)	64^c^	F							+			+	PM2, PM5, PP3	VUS-H	PM1, PM5, PP2, PP3, PS4_P, PM2_P	LPV	PM1, PM5, PP2, PP3, PP4, PM2_P	12	PV
	c.3892A>C p.(Asn1298His)	34^c^	M	11	Ascending aortic intramural hemotoma	+	6	+	2 affected				+	PM2, PP3	VUS-M	PM1, PP1, PP2, PP3, PP4, PM2_P	LPV	PM1, PP2, PP3, PP4, PM2_P	10	PV
	c.4982G>A p.(Gly1661Glu)	36^c^	F	2.61	−	+	8	+					+	PM2, PM5, PP3	VUS-H	PM1, PM5, PS4_M, PP2, PP3, PP4, PM2_P	LPV	PM1, PM5, PS4_M, PP2, PP3, PP4, PM2_P	14	PV
	c.5431G>A p.(Glu1811Lys)	30^c^	F							+			+	PM2, PS4_M, PP5	VUS-H	PM1, PS4_M, PP2, PP3	LPV	PM1, PS4_M, PP2, PP3, PP4	11	PV
		28^c^	M																	
	c.5680G>A p.(Glu1894Lys)	54^c^	M							+		+		PM2, PP3	VUS-M	PM1, PP2, PP3, PS4_P, PM2_P	LPV	PM1, PP2, PP3, PP4, PM2_P	10	PV
	c.6332G>T p.(Cys2111Phe)	35^c^	M	5.69	−	+	8	+					+	PM2, PM5, PP3	VUS-H	PM1, PM5, PP2, PP3, PP4, PM2_P	LPV	PM1, PM5, PP2, PP3, PP4, PM2_P	12	PV
	c.6380A>T p.(Asp2127Val)	57^c^	M	10.3	−	−	11	+	1 affected	+			+	PM2, PP3, PP5	VUS-H	PM1, PM5, PS4_M, PP2, PP3, PP4, PM2_P	LPV	PM1, PM5, PS4_M, PP2, PP3, PP4, PM2_P	14	PV
	c.6448C>T p.(Arg2150Cys)	58^c^	M							+			+	PM2, PP3, PS4_P	VUS-H	PM1, PS4_M, PP2, PP3	LPV	PM1, PP2, PP3, PP4, PS4_P	10	PV
	c.6490T>C p.(Cys2164Arg)	20^c^	M	5.51	−	−	8	+				+		PM1, PM2, PP5	VUS-H	PM1_S, PP2, PP3, PP4, PM2_P	LPV	PM1_S, PP2, PP3, PP4, PM2_P	12	PV
	c.6707A>G p.(Tyr2236Cys)	13^c^	F	1.72	−	+	7	−	2 affected				+^d^	PM2, PP3	VUS-M	PM1, PP1, PP2, PP3, PP4, PM2_P	LPV	PM1, PP2, PP3, PP4, PM2_P	10	PV
	c.8020T>A p.(Cys2674Ser)	40^c^	M	25.04	−	−	8	+	3 affected			+		PM1, PM2	VUS-H	PM1_S, PP1, PP2, PP3, PP4, PM2_P	LPV	PM1_S, PP2, PP3, PP4, PM2_P	12	PV
	c.8378A>G p.(Tyr2793Cys)	28^c^	M							+	+			No available records of variant assessment	VUS-U	PM1, PP2, PP3, PS4_P, PM2_P	LPV	PM1, PP2, PP3, PP4, PM2_P	10	PV
Intron	c.4460−8G>A p.(?)	51^c^	M	(45mm)	−	+	NE	+		+			+	PM4, PP1, PP3, PP5	VUS-H	PS4, PP1, PP3, PP4	LPV	PS4, PP3, PP4	10	PV

*LPV* likely pathogenic variant, *NE* not evaluated, *PV* pathogenic variant, *VUS* variant of uncertain significance, *VUS-H* variant of uncertain significance-high subclass, *VUS-M* variant of uncertain significance-mid subclass, *VUS-U* variant of uncertain significance-unsubclassified.

^a^Consistent but not highly specific phenotype definition: aortic diameter at the sinus of Valsalva above the indicated Z-score (≥2 for an individual aged ≥20 years or ≥3 for an individual aged <20 years) or aortic root dissection AND systemic score ≥7 points.

^b^Highly specific phenotype definition: aortic diameter at the sinus of Valsalva above the indicated Z-score (≥2 for an individual aged ≥20 years or ≥3 for an individual aged <20 years) or aortic root dissection AND ectopia lentis.

^c^This patient had been reported in the previous study of Samsung Medical Center [9].

^d^Highly specific phenotype was identified in a family member with the same variant. One family member with c.6707A>G p.(Tyr2236Cys) presented an aortic root Z-score of 2.31, ectopia lentis, and a systemic score of 7 points.

### Applying the new PP1/PP4 criteria

According to the new PP1/PP4 criteria, all reclassified 16 VUSs garnered stronger PP4 evidence as either the patient (*n* = 10) or a family member (*n* = 1) met the revised Ghent criteria or the literature identified a previously reported phenotype (*n* = 5) (Table 1 and Supplementary Table 3). Of five variants from the literature review, three variant c.2651G>A p.(Gly884Glu), c.4942G>A p.(Asp1648Asn), and c.5225-3C>G p.(?) were reported in patients meeting the old or revised Ghent criteria [15–17], and the other two variants c.1679G>A p.(Gly560Asp) and c.6872-14A>G p.(?) were reported in patients with highly specific phenotype [18, 19]. Subsequently, phenotype data of 16 variants were identified as highly specific phenotype (*n* = 9), consistent but not highly specific phenotype (*n* = 4), and old or revised Ghent criteria (*n* = 3). Co-segregation information did not provide additional evidence because six cases received the maximum of 5 points despite a larger number of affected family members.

Of three PVs and 26 LPVs reclassified as PVs/LPVs using the ClinGen *FBN1*-specific guideline alone, two and 15 demonstrated stronger PP4 evidence, respectively (Table 2 and Supplementary Table 3). These two PVs c.6331T>C p.(Cys2111Arg) and c.6389A>T p.(Glu2130Val) were identified in patients meeting the revised Ghent criteria and highly specific phenotype [20]. On the other hand, a female patient with c.4337A>G p.(Asp1446Gly) showed aortic root dilatation but did not meet the revised Ghent criteria. All five of her affected family members with the same variant also had aortic root dilatation only. For this patient, the PP1 instead of the PP4 criterion was applied and resulted in a maximum score of 5 points. In the 15 LPVs, PP4 was applied based on evidence that the patient (*n* = 8) or a family member (*n* = 1) met the revised Ghent criteria or previously reported phenotype data existed (*n* = 6) (Table 2). The patient with c.6380A>T p.(Asp2127Val) showed consistent but not highly specific phenotype, however, the variant was identified in a patient with highly specific phenotype in the literature [21]. Of six LPVs assigned PP4 evidence from the literature review, four were reported in patients showing highly specific phenotype, one in a patient showing consistent but not highly specific phenotype, the other one in a patient meeting Ghent criteria with no robust phenotype data [15, 22–27]. Subsequently, phenotype data of 15 LPVs were identified as highly specific phenotype (*n* = 11), consistent but not highly specific phenotype (*n* = 3), and old or revised Ghent criteria (*n* = 1).

### Representative cases

The patient with c.2504A>T p.(Glu835Val) met the revised Ghent criteria with aortic root dilatation (Z-score of 2.95), bilateral ectopia lentis, and a systemic score of 12 points. She had no family history of sudden cardiac death or aortic dissection. Her parents did not show clinical features of MFS, but *FBN1* testing was not performed. Based on the ClinGen *FBN1*-specific guideline, PP2, PP4, and PM2_supporting criteria were applied for the c.2504A>T p.(Glu835Val) variant, which allowed VUS classification. However, upon applying the new PP1/PP4 criteria, this variant was awarded 7 points (1 point for PP2, 5 points for PP4, and 1 point for PM2_supporting) and was reclassified as an LPV.

**Fig. 2:**
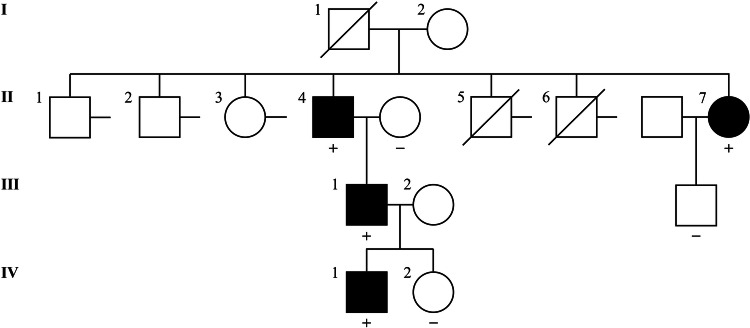
Pedigree of a family with Marfan syndrome and the *FBN1* c.47T>C p.(Leu16Pro) variant. The results of individuals who underwent genetic testing for *FBN1* are indicated using either “+” (variant detected) or “−” (variant not detected).

In cases with an affected family member, the new PP1/PP4 criteria were applied as follows. The blood sample of a patient with suspected MFS was requested for *FBN1* sequencing from an external medical institution. Clinical information was not provided in detail, except for the pedigree (Fig. 2). The patient (II:7) was revealed to have a variant of c.47T>C p.(Leu16Pro) and three affected family members. Her brother (II:4) had the same variant and had undergone the Bentall operation for a dilated aortic root (Z-score 10.03) with severe aortic regurgitation. However, he (II:4) did not have ectopia lentis and received a systemic score of 5 points. His son (III:1) and grandson (IV:1) had the same variant. His son (III:1) fulfilled the revised Ghent criteria with aortic root dilatation (Z-score 8.38), bilateral ectopia lentis, and a systemic score of 4 points. The grandson (IV:1) was 6 years old and had mild annuloaortic ectasia with a Z-score of 2.72, no ectopia lentis, and a systemic score of 1 point. Because her nephew (III:1) met the revised Ghent criteria, PP4 evidence could be applied for the variant. According to the ClinGen *FBN1*-specific guideline, PP1, PP2, PP4, and PM2_supporting criteria resulted in its classification as a VUS. However, after applying the new PP1/PP4 criteria, the variant was awarded 7 points (PP2, PP4, and PM2_supporting) and was reclassified as an LPV.

On the other hand, there was an equivocal case for applying the new PP1/PP4 criteria. The patient with c.6403G>A p.(Asp2135Asn) met the revised Ghent criteria with a high aortic root Z-score of 7.93 and a systemic score of 7 points, allowing application of the PP4 criterion. The *FBN1* variant was awarded 7 points (PP2, PP4, and PM2_supporting) and was reclassified as an LPV. However, she was found to have mosaic Turner syndrome. Variants of chromosome Y–related genes were detected on NGS-MFS and fluorescence in situ hybridization (FISH) confirmed 48.8% of 500 cells with monosomy X, 29.0% with XY, and 22.2% with chromosome X and a pseudo-isodicentric chromosome Y. Chromosomal analysis of 30 cultured cells presented a mosaic pattern with 45,X[17]/46,X,psu idic(Y)(q11.23)[13]. She demonstrated a bicuspid aortic valve, aortic root dilatation, relative narrowing of the aortic isthmus, and partial anomalous pulmonary venous return. Ectopia lentis was not present. She underwent the Bentall operation and gonadectomy and has been followed since, with ongoing management of the cardiovascular complications of Turner syndrome.

### Reclassification results of VUS subclasses

**Fig. 3:**
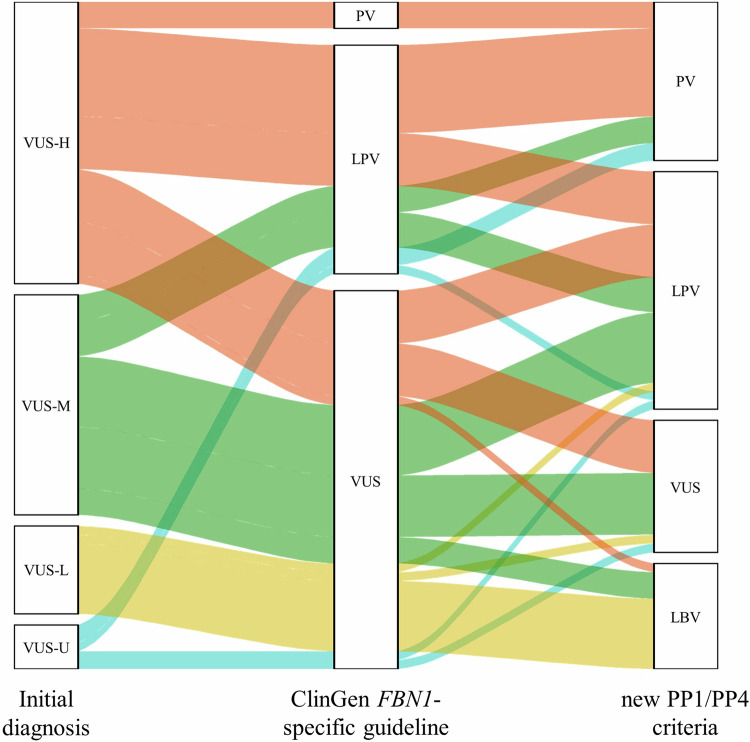
Reclassification of VUS subclasses according to ClinGen *FBN1*-specific guideline with new PP1/PP4 criteria. LBV likely benign variant, LPV likely pathogenic variant, PV pathogenic variant, VUS variant of uncertain significance, VUS-H variant of uncertain significance-high subclass, VUS-L variant of uncertain significance-low subclass, VUS-M variant of uncertain significance-mid subclass, VUS-U variant of uncertain significance-unsubclassified.

A total of 72 VUSs were subclassified as 32 VUS-Hs, 25 VUS-Ms, 10 VUS-Ls, and five VUS-Us, respectively (Fig. 3 and Supplementary Table 3). Final reclassification tiers of VUS-Hs were 13 PVs, 12 LPVs, six VUSs, and one LBV. Final reclassification tiers of VUS-Ms were three PVs, 12 LPVs, seven VUSs, and three LBVs. Final reclassification tiers of VUS-Ls were one LPV, one VUS, and eight LBVs. VUS-H had the highest reclassification rate to PV/LPV (78.1%), followed by VUS-M (60.0%) and VUS-L (10%).

## Discussion

The new PP1/PP4 criteria provide specific information of PP4 application and facilitated a significant increase in the reclassification rate, which resulted in 37.2% (16/43) of remaining VUSs in the present study being reclassified as PVs/LPVs. Using this approach, a variant detected in a patient meeting the revised Ghent criteria and that was highly suspected to be causative was reclassified as a PV/LPV. This is mainly possible due to the weighted strength of PP4, as a variant awarded PP4 of 5 points can be classified as a PV/LPV with at least one additional pathogenic evidence. Overall, using the ClinGen *FBN1*-specific guideline and the new PP1/PP4 criteria, 62.5% (45/72) of VUSs were successfully reassessed as PVs/LPVs. When subclassifying VUSs, VUS-H showed the highest reclassification rate to PV/LPV in line with the previous study [14]. In contrast, one VUS-L was reclassified as an LPV although none were reclassified as PV/LPV in the previous study [14]. This variant was c.6403G>A p.(Asp2135Asn) that was detected in the patient with mosaic Turner syndrome meeting the revised Ghent criteria. Twelve variants were reclassified as LBVs according to the points-based system; however, we regarded them as VUSs based on the ClinGen *FBN1*-specific guideline because the applied evidence is conflicting or insufficient before additional functional or phenotype data are available to evaluate the pathogenicity.

For successful application of the PP4 criterion, a full workup of clinical manifestations of the patient with suspected MFS is necessary [11]. However, clinical laboratories do not always have access to robust clinical data of the patient, especially those from external institutions [28]. In this study, the evaluation of clinical information was restricted because only blood samples and limited clinical information were provided by the referring institutions, and the details of full patient evaluations were not available in many cases. If sufficient medical history is available for application of the revised Ghent criteria, a larger number of VUSs might be reclassified as PVs/LPVs. Therefore, cooperation and communication among clinicians would be helpful for accurate assessment of variants. Laboratorial reports of genetic testing can also be improved to inform clinicians more detailed and personalized recommendations regarding how a VUS can be reclassified by providing additional clinical information that eventually shows fulfillment of the Ghent criteria.

In this study, we applied the PP4 criterion to five VUSs based on previous literature reports (Table 1). Among them, the c.4942G>A p.(Asp1648Asn) variant reported in a patient meeting the old Ghent criteria was scored 5 points because the aforementioned study was performed before the Ghent nosology was revised [16]. Several comparison studies have demonstrated excellent 91% correlation of positive rates of MFS patients meeting the old Ghent criteria and revised Ghent criteria [16, 29–31]. In addition, in a follow-up study, some MFS patients developed further clinical manifestations, including aortic pathology, ectopia lentis, and other systemic features [31]. In line with that previous study, the Z-score of a present 10-year-old patient with c.6704G>T p.(Gly2235Val) increased from 2.37 to 3.00 on follow-up measurement. This shows that a patient initially not fulfilling the revised Ghent criteria might present additional clinical symptoms and meet the criteria in a future visit, allowing application of the PP4 criterion and the re-designation of a VUS to a PV/LPV. Considering the progression of the disease, clinical manifestations should be reassessed at regular intervals, especially in pediatric patients [31, 32].

Application of the PP4 criterion was very useful in assessing *FBN1* variants. Nevertheless, the increasing use of this criterion has limitations. First, there is a possibility of redundant application of pathogenic evidence. For example, as noted in the new PP1/PP4 criteria, PP4 evidence and PS4 case-counting evidence can overlap, resulting in overvaluation of pathogenicity [11]. The new PP1/PP4 criteria suggested that addition of PS4 evidence to the PP1/PP4 evidence be capped at 9 points to prevent results above the upper limit of the LPV score range. Based on this, a previously observed proband should not be considered in both PP4 and PS4 evaluations. Given this recommendation, the probands who met the Ghent criteria and were considered in the PP4 data were not included in the PS4 data to evaluate VUSs.

Second, although fulfillment of the Ghent criteria is essential and highly specific for MFS, including a patient meeting the Ghent criteria to obtain PP4 evidence can result in incorrect assignment of a variant as pathogenic, especially in cases with other diseases showing a similar phenotype. For example, a previous patient with Loeys-Dietz syndrome type 3 with a *SMAD3* LPV fulfilled both the old and revised Ghent criteria [31]. There are also reports that three patients with *TGFBR2* variants fulfilled the diagnostic criteria for both Loeys-Dietz syndrome and MFS, and one patient who had been diagnosed with MFS was diagnosed with homocystinuria with compound heterozygous variants in the *CBS* gene on a targeted multigene panel test [33, 34]. In the present study, a patient with mosaic Turner syndrome and the *FBN1* variant c.6403G>A p.(Asp2135Asn) met the revised Ghent criteria with an aortic root Z-score of 7.93 and a systemic score of 7 points. After full evaluation of the patient’s clinical features, such as a bicuspid aortic valve and partial anomalous pulmonary venous return, the patient was thought to have a complication of Turner syndrome rather than MFS. Further evidence including functional or phenotype data is needed to confirm the pathogenicity of this variant. Because a wide spectrum of genetic diseases involve the cardiovascular system, including Loeys-Dietz syndrome and Turner syndrome [1, 35], the possibility of misdiagnosis must be excluded through both clinical evaluation and genetic testing.

This finding suggests that down-grading PP4 strength or full evaluation of other loci should be considered when a patient does not meet the highly specific phenotype for MFS despite meeting the revised Ghent criteria. To preclude abuse of stronger PP4 evidence, application only if a variant is identified in a patient presenting highly specific phenotype could be suggested. In this study, when stronger PP4 was applied for highly specific phenotype, nine VUSs were reclassified as LPVs, but it was still statistically significant. On the other hand, when a phenotype was consistent but not highly specific, application of stronger PP4 should be preceded by excluding the possible PV/LPV of other loci through multi-gene panel test and/or clinical evaluation since NGS-MFS was able to identify the main genetic cause of aortopathy in the patient with mosaic Turner syndrome. Of seven variants with consistent but not highly specific phenotype, four variants were detected on NGS (Supplementary Table 3). Unless clinical and genetic investigation is sufficient, it might be more plausible to apply PP4 criterion with lower strength.

Finally, there is a possibility of an actual causative variant existing in the deep intronic region, which most clinical laboratories do not routinely test or report [36]. A previous study reported two deep intronic variants in two unrelated probands with MFS fulfilling the revised Ghent criteria [36]. No PVs/LPVs were identified using Sanger sequencing or multiplex ligation-dependent probe amplification of *FBN1* and/or panel-based NGS. With whole-genome sequencing and RNA analysis, *FBN1* variants c.6163+1484A>T and c.5788+36C>A were detected and confirmed to be pathogenic, i.e., able to induce aberrant mRNAs. If such individuals had a variant in coding exons or flanking sequences of *FBN1*, the variant could be incorrectly defined as pathogenic by adding PP4 evidence, and further evaluation would not be performed. Because a presumed *CFTR* PV c.443T>C p.(Ile148Thr) was later found to be in linkage disequilibrium with an actual causative variant c.3067_3072del p.(Ile1023_Val1024del) and was reclassified as benign, linkage disequilibrium of an *FBN1* deep intronic variant should be considered [11]. As such, the limitations of the new PP1/PP4 criteria must be considered.

In summary, we present a reassessment of *FBN1* VUSs collected over nine years according to the ClinGen *FBN1*-specific guideline and new PP1/PP4 criteria. Before applying the new PP1/PP4 criteria, phenotypic data should be assessed to eliminate the risk of overestimation. This investigation significantly improved the reclassification rate of VUSs to PVs/LPVs and provided useful diagnostic evidence. In conclusion, the new PP1/PP4 criteria will be helpful to reclassify highly suspicious VUSs detected in MFS patients fulfilling the revised Ghent criteria.

## Supplementary information

Supplementary Figure 1

Supplementary Table 1

Supplementary Table 2

Supplementary Table 3

## Data Availability

The data that support the findings of this study are available on request from the corresponding authors.

## References

[1] Zucker EJ. Syndromes with aortic involvement: pictorial review. Cardiovasc Diagn Ther. 2018;8:S71–81. 29850420 10.21037/cdt.2017.09.14 PMC5949600

[2] Loeys BL, Dietz HC, Braverman AC, Callewaert BL, De Backer J, Devereux RB, et al. The revised Ghent nosology for the Marfan syndrome. J Med Genet. 2010;47:476–85. 20591885 10.1136/jmg.2009.072785

[3] Baetens M, Van Laer L, De Leeneer K, Hellemans J, De Schrijver J, Van De Voorde H, et al. Applying massive parallel sequencing to molecular diagnosis of Marfan and Loeys-Dietz syndromes. Hum Mutat. 2011;32:1053–62. 21542060 10.1002/humu.21525

[4] Meester JAN, Peeters S, Van Den Heuvel L, Vandeweyer G, Fransen E, Cappella E, et al. Molecular characterization and investigation of the role of genetic variation in phenotypic variability and response to treatment in a large pediatric Marfan syndrome cohort. Genet Med. 2022;24:1045–53. 35058154 10.1016/j.gim.2021.12.015 PMC9680912

[5] De Paepe A, Devereux RB, Dietz HC, Hennekam RC, Pyeritz RE. Revised diagnostic criteria for the Marfan syndrome. Am J Med Genet. 1996;62:417–26. 8723076 10.1002/(SICI)1096-8628(19960424)62:4<417::AID-AJMG15>3.0.CO;2-R

[6] Richards S, Aziz N, Bale S, Bick D, Das S, Gastier-Foster J, et al. Standards and guidelines for the interpretation of sequence variants: a joint consensus recommendation of the American College of Medical Genetics and Genomics and the Association for Molecular Pathology. Genet Med. 2015;17:405–24. 25741868 10.1038/gim.2015.30 PMC4544753

[7] Rivera-Muñoz EA, Milko LV, Harrison SM, Azzariti DR, Kurtz CL, Lee K, et al. ClinGen Variant Curation Expert Panel experiences and standardized processes for disease and gene-level specification of the ACMG/AMP guidelines for sequence variant interpretation. Hum Mutat. 2018;39:1614–22. 30311389 10.1002/humu.23645 PMC6225902

[8] *FBN1* variant curation expert panel. Clingen *FBN1* expert panel specifications to the ACMG/AMP variant interpretation guidelines version 1. 2022. Available: https://clinicalgenome.org/site/assets/files/7445/clingen_fbn1_acmg_specifications_v1.pdf. Accessed 17 May 2024.

[9] Yoon E, Lee JK, Park TK, Chang SA, Huh J, Kim JW, et al. Experience of reassessing *FBN1* variants of uncertain significance by gene-specific guidelines. J Med Genet. 2023;61:57–60. 37558401 10.1136/jmg-2023-109433

[10] Kim SW, Kim B, Kim Y, Lee KA. Re-evaluation of a Fibrillin-1 Gene Variant of Uncertain Significance Using the ClinGen Guidelines. Ann Lab Med. 2024;44:271–78. 37840311 10.3343/alm.2023.0152 PMC10813823

[11] Biesecker LG, Byrne AB, Harrison SM, Pesaran T, Schäffer AA, Shirts BH, et al. ClinGen guidance for use of the PP1/BS4 co-segregation and PP4 phenotype specificity criteria for sequence variant pathogenicity classification. Am J Hum Genet. 2024;111:24–38. 38103548 10.1016/j.ajhg.2023.11.009 PMC10806742

[12] Tavtigian SV, Harrison SM, Boucher KM, Biesecker LG. Fitting a naturally scaled point system to the ACMG/AMP variant classification guidelines. Hum Mutat. 2020;41:1734–7. 32720330 10.1002/humu.24088 PMC8011844

[13] Kim JA, Kwon WK, Kim JW, Jang JH. Variation spectrum of *MECP2* in Korean patients with Rett and Rett-like syndrome: a literature review and reevaluation of variants based on the ClinGen guideline. J Hum Genet. 2022;67:601–6. 35606502 10.1038/s10038-022-01044-x

[14] Bennett G, Karbassi I, Chen W, Harrison SM, Lebo MS, Meng L, et al. Distinct rates of VUS reclassification are observed when subclassifying VUS by evidence level. Genet Med. 2025;28:101400. 10.1016/j.gim.2025.101400 PMC12181064 40035215

[15] Attanasio M, Lapini I, Evangelisti L, Lucarini L, Giusti B, Porciani M, et al. *FBN1* mutation screening of patients with Marfan syndrome and related disorders: detection of 46 novel *FBN1* mutations. Clin Genet. 2008;74:39–46. 18435798 10.1111/j.1399-0004.2008.01007.x

[16] Lerner-Ellis JP, Aldubayan SH, Hernandez AL, Kelly MA, Stuenkel AJ, Walsh J, et al. The spectrum of *FBN1*, *TGFβR1*, *TGFβR2* and *ACTA2* variants in 594 individuals with suspected Marfan Syndrome, Loeys-Dietz Syndrome or Thoracic Aortic Aneurysms and Dissections (TAAD). Mol Genet Metab. 2014;112:171–6. 24793577 10.1016/j.ymgme.2014.03.011

[17] Kim KH, Kim TY, Kim SJ, Cho YG, Park J, Jang W. Targeted Panel Sequencing Identifies an Intronic c.5225-3C>G Variant of the *FBN1* Gene Causing Sporadic Marfan Syndrome with Annuloaortic Ectasia. Genes. 2022;13:2108. 36421783 10.3390/genes13112108 PMC9690865

[18] Baudhuin LM, Kotzer KE, Lagerstedt SA. Increased frequency of *FBN1* truncating and splicing variants in Marfan syndrome patients with aortic events. Genet Med. 2015;17:177–87. 25101912 10.1038/gim.2014.91

[19] Muiño-Mosquera L, Steijns F, Audenaert T, Meerschaut I, De Paepe A, Steyaert W, et al. Tailoring the American College of Medical Genetics and Genomics and the Association for Molecular Pathology Guidelines for the Interpretation of Sequenced Variants in the *FBN1* Gene for Marfan Syndrome: Proposal for a Disease- and Gene-Specific Guideline. Circ Genom Precis Med. 2018;11:e002039. 29875124 10.1161/CIRCGEN.117.002039

[20] Duan DM, Chiu HH, Chen PL, Yeh PT, Yu CW, Yang KC, et al. Clinical manifestations and genetic characteristics in the Taiwan thoracic aortic aneurysm and dissection cohort - a prospective cohort study. J Formos Med Assoc. 2022;121:1093–101. 34456093 10.1016/j.jfma.2021.08.016

[21] Evangelisti L, Lucarini L, Attanasio M, Lapini I, Giusti B, Porciani C, et al. A single heterozygous nucleotide substitution displays two different altered mechanisms in the *FBN1* gene of five Italian Marfan patients. Eur J Med Genet. 2010;53:299–302. 20538085 10.1016/j.ejmg.2010.06.002

[22] Guo D, Jin G, Zhou Y, Zhang X, Cao Q, Lian Z, et al. Mutation spectrum and genotype-phenotype correlations in Chinese congenital ectopia lentis patients. Exp Eye Res. 2021;207:108570. 33844962 10.1016/j.exer.2021.108570

[23] Guo D, Li S, Xiao X, Jiang Y, Wang Y, Jin G, et al. Clinical and Genetic Landscape of Ectopia Lentis Based on a Cohort of Patients From 156 Families. Invest Ophthalmol Vis Sci. 2024;65:20. 38190127 10.1167/iovs.65.1.20 PMC10777873

[24] Cho JS, Park J, Kwon JB, Kim DW, Park MW. 3D Printed Personalized External Aortic Root Model in Marfan Syndrome with Isolated Sinus of Valsalva Aneurysm Caused by a Novel Pathogenic *FBN1* p.Gly1127Cys Variant. Diagnostics. 2021;11:1057. 34201307 10.3390/diagnostics11061057 PMC8227084

[25] Comeglio P, Johnson P, Arno G, Brice G, Evans A, Aragon-Martin J, et al. The importance of mutation detection in Marfan syndrome and Marfan-related disorders: report of 193 *FBN1* mutations. Hum Mutat. 2007;28:928. 17657824 10.1002/humu.9505

[26] Mannucci L, Luciano S, Salehi LB, Gigante L, Conte C, Longo G, et al. Mutation analysis of the FBN1 gene in a cohort of patients with Marfan Syndrome: A 10-year single center experience. Clin Chim Acta. 2020;501:154–64. 31730815 10.1016/j.cca.2019.10.037

[27] Tjeldhorn L, Rand-Hendriksen S, Gervin K, Brandal K, Inderhaug E, Geiran O, et al. Rapid and efficient FBN1 mutation detection using automated sample preparation and direct sequencing as the primary strategy. Genet Test. 2006;10:258–64. 17253931 10.1089/gte.2006.258-264

[28] Basel-Salmon L. Phenotypic compatibility and specificity in genomic variant classification. Eur J Hum Genet. 2024;32:471–3. 38351291 10.1038/s41431-024-01554-6 PMC11061282

[29] Radonic T, de Witte P, Groenink M, de Bruin-Bon RA, Timmermans J, Scholte AJ, et al. Critical appraisal of the revised Ghent criteria for diagnosis of Marfan syndrome. Clin Genet. 2011;80:346–53. 21332468 10.1111/j.1399-0004.2011.01646.x

[30] Yang JH, Han H, Jang SY, Moon JR, Sung K, Chung TY, et al. A comparison of the Ghent and revised Ghent nosologies for the diagnosis of Marfan syndrome in an adult Korean population. Am J Med Genet A. 2012;158A:989–95. 22162372 10.1002/ajmg.a.34392

[31] Vanem TT, Böker T, Sandvik GF, Kirkhus E, Smith HJ, Andersen K, et al. Marfan syndrome: Evolving organ manifestations-A 10-year follow-up study. Am J Med Genet A. 2020;182:397–408. 31825148 10.1002/ajmg.a.61441

[32] Faivre L, Collod-Beroud G, Adès L, Arbustini E, Child A, Callewaert BL, et al. The new Ghent criteria for Marfan syndrome: what do they change? Clin Genet. 2012;81:433–42. 21564093 10.1111/j.1399-0004.2011.01703.x

[33] Attias D, Stheneur C, Roy C, Collod-Béroud G, Detaint D, Faivre L, et al. Comparison of clinical presentations and outcomes between patients with *TGFBR2* and *FBN1* mutations in Marfan syndrome and related disorders. Circulation. 2009;120:2541–9. 19996017 10.1161/CIRCULATIONAHA.109.887042

[34] Qin M, Zhu X, Zhang Z, Li X, Yan Z, Wang Y, et al. Genetic analysis and preimplantation genetic diagnosis of Chinese Marfan syndrome patients. J Genet Genomics. 2019;46:319–23. 31279624 10.1016/j.jgg.2019.04.003

[35] Thunström S, Thunström E, Naessén S, Berntorp K, Laczna Kitlinski M, Ekman B, et al. Aortic size predicts aortic dissection in Turner syndrome - A 25-year prospective cohort study. Int J Cardiol. 2023;373:47–54. 36410543 10.1016/j.ijcard.2022.11.023

[36] Kim JA, Jang MA, Jang SY, Kim DK, Kim YG, Kim JW, et al. Overcoming challenges associated with identifying *FBN1* deep intronic variants through whole-genome sequencing. J Clin Lab Anal. 2024;38:e25009. 38234087 10.1002/jcla.25009 PMC10829686

