# Supplementary figures and images for: Reassessment of FBN1 variants of uncertain significance using updated ClinGen guidance for PP1/BS4 and PP4 criteria
Source: Eur J Hum Genet. 2025 Apr 1;33(5):666–74. doi: 10.1038/s41431-025-01826-9 (PMC12048594; doi:10.1038/s41431-025-01826-9)

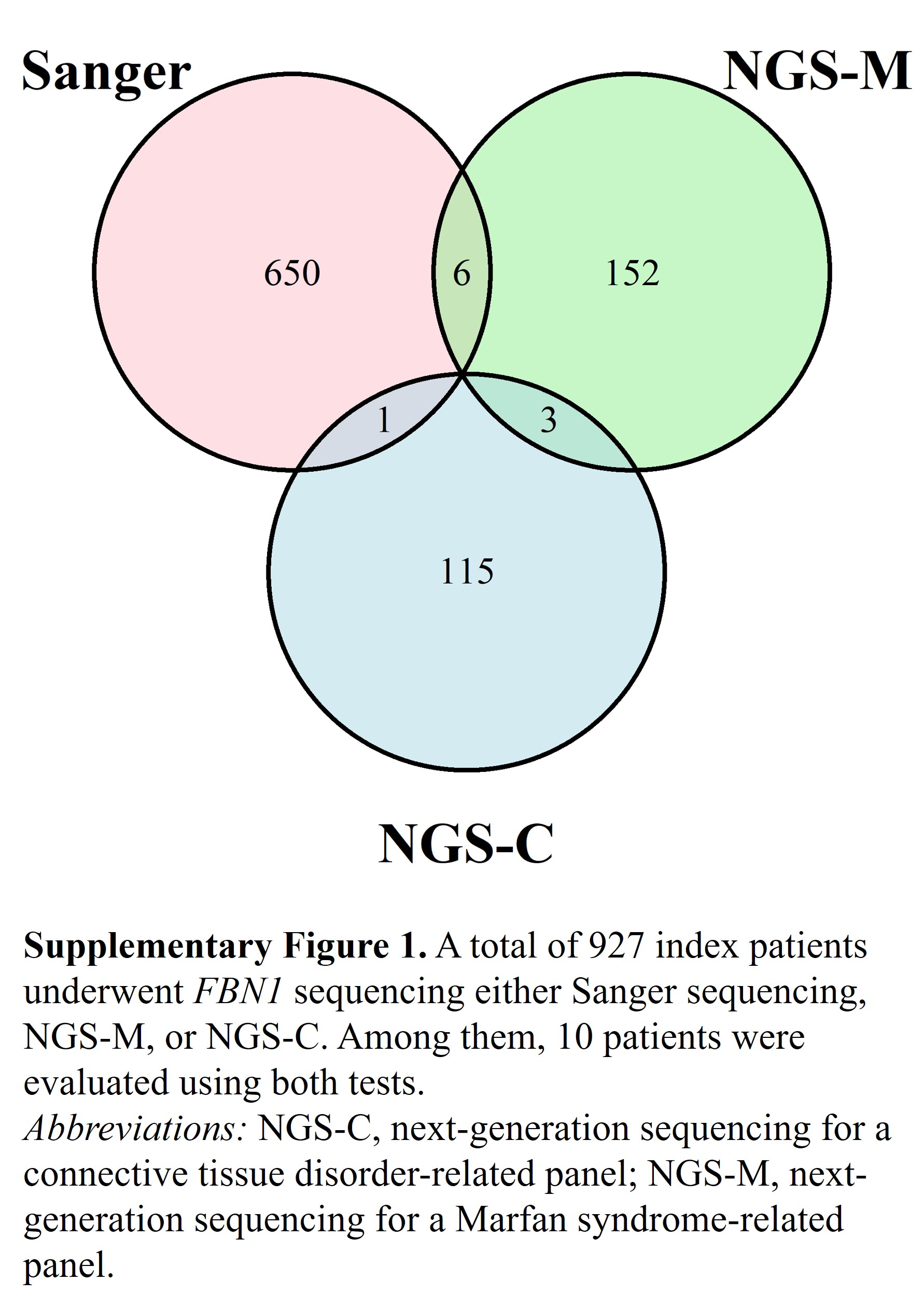

Supplement: Supplementary file 1 — Supplementary Figure 1 [file 41431_2025_1826_MOESM1_ESM.jpg]
